# 1175. Influenza Vaccine Hesitancy in Hospitalized Children, Before and During the COVID-19 Pandemic

**DOI:** 10.1093/ofid/ofab466.1368

**Published:** 2021-12-04

**Authors:** Marisa Orbea, Jose Dominguez, Rachel Cunningham, C Mary Healy, Julie A Boom, Claire Bocchini

**Affiliations:** 1 Baylor College of Medicine, Texas Children’s Hospital, Houston, Texas; 2 Cambridge Health Alliance, Harvard Medical School, Cambridge, Massachusetts; 3 Texas Children’s Hospital, Houston, Texas; 4 Baylor College of Medicine, Houston, TX

## Abstract

**Background:**

Influenza vaccine is recommended for all children ≥6 months, yet uptake is suboptimal. We aimed to quantify child influenza vaccine coverage and identify factors associated with influenza vaccine hesitancy (VH) before and during the COVID-19 pandemic.

**Methods:**

We conducted a prospective, repeated cross-sectional assessment in English and Spanish of caregiver influenza knowledge, attitudes, behaviors, and associated VH among hospitalized children 6 months through 18 years at a large pediatric medical institution. Caregivers were enrolled 4-5 days per week, between 12/11/2019--1/31/2020 and 12/8/2020--4/5/2021. VH was assessed using the Parent Attitudes about Childhood Vaccines (PACV) survey; PACV score ≥50 denoted VH. Descriptive statistics and multivariable logistic regression were used.

**Results:**

During 2019-2020 and 2020-2021 influenza seasons, 269/282 (95%) and 295/307 (96%) of approached caregivers enrolled, respectively. By caregiver report, 94% of children in 2019-2020 and 91% in 2020-2021 were up-to-date with routine childhood vaccines (p=0.13). Specific to influenza vaccine, 73% and 68% of children received or planned to receive influenza vaccine in 2019-2020 and 2020-2021, respectively (p=0.13). Based on PACV score, 13% of parents were VH in 2019-2020 compared with 17% in 2020-2021 (p=0.24; Figure 1).

Caregivers who had not/did not intend to vaccinate their children had a higher family income (71% vs. 57% >&30,000, p< 0.01) and were less likely to be Hispanic/Latino (35% vs. 47%, p=0.02). 77% of caregivers were satisfied with information about influenza vaccine received from healthcare providers. Overall, 36% believed “you can get the flu from the flu shot.” In 2020-2021, caregivers were less likely to believe that “flu can be a dangerous infection in children,” to be “scared of my child getting the flu” and to agree that “all children over 6 months of age should receive the flu shot every year” (Table 1).

Table 1. Caregiver knowledge and attitudes about seasonal influenza vaccine, 2019-20 versus 2020-21

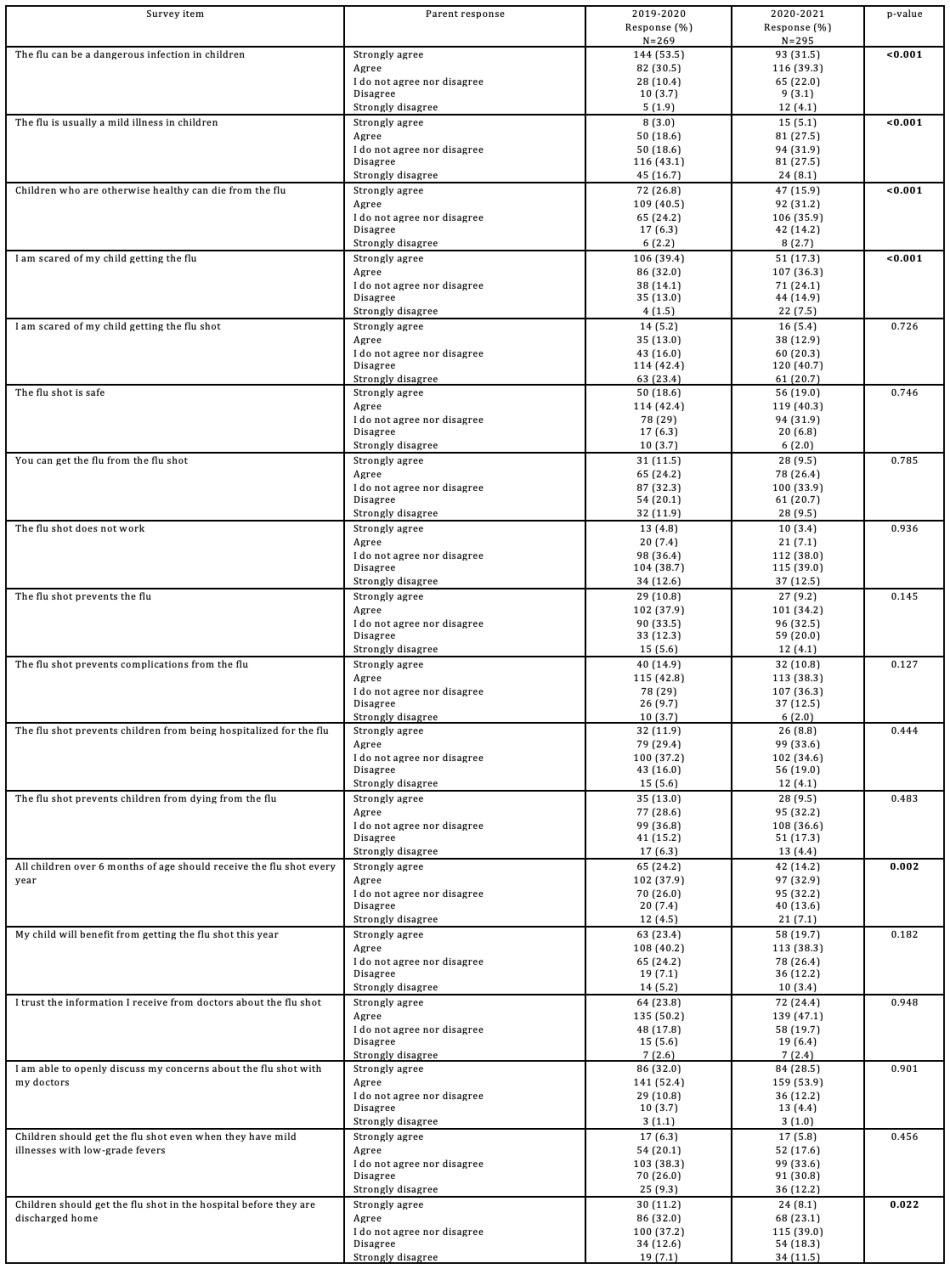

Figure 1. Influenza vaccine uptake by PACV score during 2019-2020 (a) and 2020-2021 (b) seasons

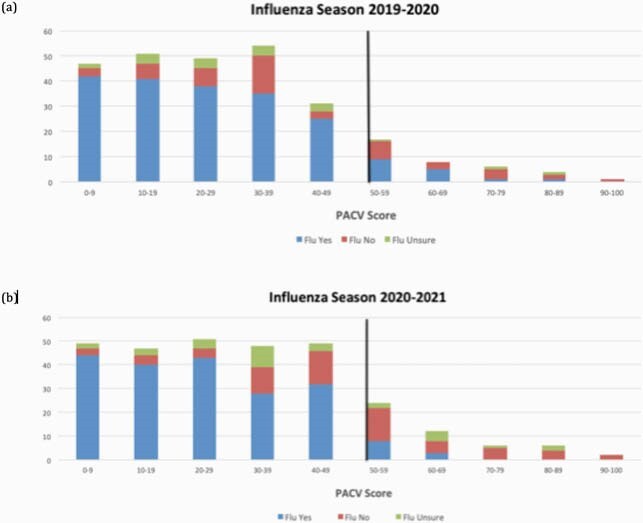

**Conclusion:**

During the COVID-19 pandemic, caregivers of hospitalized children were less concerned about influenza than pre-pandemic and misinformation about influenza and influenza vaccine persisted. Increased efforts may be needed to educate caregivers about the importance of influenza immunization during the 2021-22 season.

**Disclosures:**

**C. Mary Healy, MD**, **Dexcom** (Shareholder)**Intuitive** (Shareholder)**Quidel Corporation** (Shareholder)**Up to Date** (Other Financial or Material Support, Honorarium)**Vapotherm** (Shareholder)

